# Integrated microwave-assisted acetic acid pretreatment and endoglucanase-xylanase hydrolysis for nanocrystalline cellulose preparation^☆^

**DOI:** 10.1016/j.mex.2026.103961

**Published:** 2026-05-20

**Authors:** K.Y. Lim, K.Y. Foo

**Affiliations:** River Engineering and Urban Drainage Research Centre (REDAC), Universiti Sains Malaysia, Tuanku Syed Sirajuddin Engineering Campus, Seri Ampangan, 14300 Nibong Tebal, Penang, Malaysia

**Keywords:** Acetic acid, Endoglucanase, Enzymatic hydrolysis, Microwave, Nanocrystalline cellulose, Organosolv pretreatment, Tangerine peel, Xylanase

## Abstract

This study presents a high-efficiency extraction strategy for nanocrystalline cellulose (NCC) from tangerine peel as a model lignocellulosic biomass, using sequential microwave (MW)-assisted acetic acid (AA) pretreatment and dual-enzyme (endoglucanase and xylanase) hydrolysis. MW-assisted AA pretreatment (50 % AA, 100 °C, 10 min) achieves rapid volumetric heating and enhanced solvent penetration, and selective removal of hemicellulose, lignin and pectin components, while preserving crystalline cellulose domains, generating a highly accessible substrate for enzymatic processing. Subsequent hydrolysis with endoglucanase and xylanase (25 U/g substrate (TP-CFs) for endoglucanase and 50 U/g substrate (TP-CFs) for xylanase, 50 °C, 5 h, pH 4.8) depolymerizes amorphous cellulose regions and residual hemicellulosic barriers under mild conditions, resulting in NCC with uniform nano-fibrillar morphology (length 603.48 nm, diameter 9.23 nm, aspect ratio 65.36), high crystallinity (64.35 %), enhanced thermal stability (T_max_ 338.9 °C), colloidal stability (−30.2 mV) and high production yield (70.8 %). The MW-AA and dual-enzyme platform reduces chemical usage, lowers energy demand and enables selective biomass fractionation, establishing a simple, scalable and environmentally friendly pathway for high-performance nanocellulose preparation.

• Microwave-assisted acetic acid pretreatment promotes rapid, selective biomass fractionation and cellulose preservation

• Dual endoglucanase-xylanase synergy drives efficient amorphous cellulose removal under mild reaction conditions

• The hybrid platform delivers high-yield nanocellulose production and structural integrity

## Graphical abstract

**Figure:**
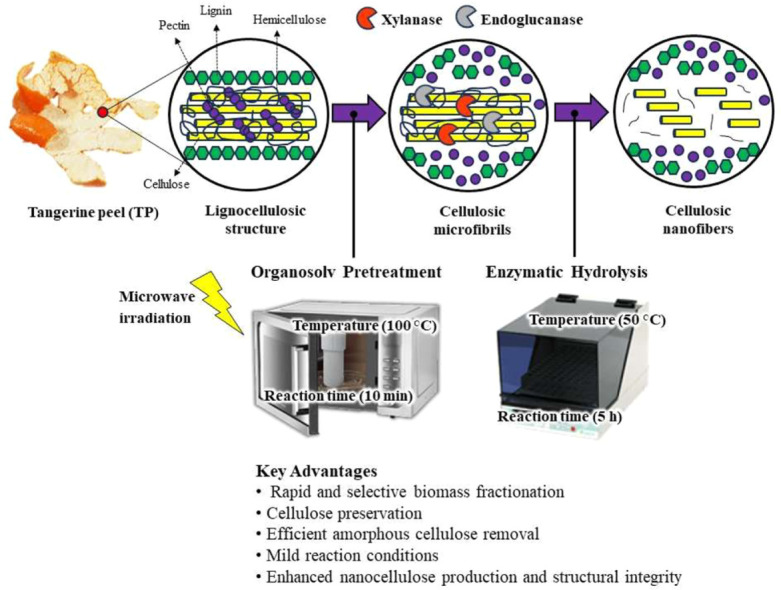

## Specifications table

**Table:** 

**Subject area**	Materials Science
**More specific subject area**	Green synthesis technique, Nanomaterial and Resource recovery
**Name of your method**	Integrated Microwave-Assisted Acetic Acid Pretreatment and Endoglucanase-Xylanase Hydrolysis for Nanocrystalline Cellulose Preparation
**Name and reference of original method**	1. K.Y. Lim, K.Y. Foo, Synthesis of nanocrystalline cellulose via microwave-assisted acetic acid pretreatment and endoglucanase-xylanase-mediated enzymatic hydrolysis, Scientific Reports (2026). https://doi.org/10.1038/s41598-026-53753-4. 2. K.Y. Lim, K.Y. Foo, Facile synthesis of nanocrystalline cellulose from rice husk by microwave heating: Evaluation of morphological architectures from the macro-to-nano dimensions, Cellulose 31 (2024) 9661–9679. 3. L.D.S.C. de Carvalho, R.G.R. Brenes, M.A. Grieco, N. Bojorge, N. Pereira Jr, Production of cellulose nano/microfibers through simultaneous milling and enzymatic hydrolysis with an optimized cocktail of cellulase/xylanase/LPMO, Industrial Crops and Products 220 (2024) 119,137.
**Resource availability**	-

## Background

Nanocellulose has emerged as a highly versatile material in advanced materials science due to its renewable origin, environmental compatibility, low density, outstanding mechanical strength, tunable surface chemistry, and remarkable optical and barrier properties. These attributes position nanocellulose as a promising candidate for a wide range of applications, including composites, packaging, biomedical materials and environmental remediation [1]. Lignocellulosic biomass, being abundant and renewable, provides a strategic platform for sustainable nanocellulose production and valorization of agricultural and industrial residues. However, its complex hierarchical structure, in which cellulose microfibrils are tightly embedded within a rigid matrix of lignin and hemicellulose, severely limits the efficiency, selectivity and controllability of nanocellulose extraction [2]. A wide range of approaches has been developed for the isolation of nanocellulose, broadly categorized into chemical, physical and biological methods. Conventional chemical techniques, particularly strong acid hydrolysis using sulfuric acid, remain among the most widely employed methods for the preparation of nanocrystalline cellulose. However, these methods often suffer from poor process controllability, excessive degradation of cellulose chains, generation of hazardous waste, and compromised thermal stability and surface properties of the resulting nanocellulose [3]. Alternatively, oxidative and alkali-based treatments have been explored, but these approaches similarly involve intensive chemical usage and multi-step purification processes, which increase the environmental burden and operational complexity.

Physical methods, including high-speed homogenization, microfluidization, high-pressure homogenization, ultrasonication and mechanical refining, have also been extensively applied to produce nanocellulose, particularly cellulose nanofibers. These techniques rely on the application of intense mechanical forces to disintegrate fiber structures into nanoscale fibrils. While effective in isolating fibrillated structures with high aspect ratios, physical methods are typically energy-intensive, require specialized equipment, and often necessitate prior chemical or enzymatic pretreatment to reduce energy demand and improve process efficiency [4]. As a result, their scalability and economic feasibility remain constrained. Biological approaches, specifically enzymatic hydrolysis and fermentation-based processes, have gained aesthetic attention as environmentally benign alternatives. Enzymatic hydrolysis offers high selectivity under mild operating conditions, and enables controlled depolymerization of lignocellulosic substrates, while preserving the crystalline domains of cellulose. Fermentation-assisted processes further enhance biomass accessibility through microbial degradation of lignin and hemicellulose components. Nevertheless, these biological routes are often limited by slow reaction kinetics, high enzyme cost, sensitivity to process conditions, and challenges associated with enzyme recovery and reuse, which can hinder large-scale implementation [5].

Given these limitations, hybrid strategies that integrate multiple treatment approaches have emerged as promising solutions to balance efficiency, selectivity and sustainability. In particular, organosolv pretreatment using acetic acid (AA) has been recognized for its ability to selectively solubilize lignin and partially remove hemicellulose, without compromising the cellulose integrity [6]. The incorporation of microwave (MW)-assisted heating further enhances this process by supporting rapid and uniform volumetric heating, improved solvent penetration, and accelerated biomass deconstruction [7]. This combination produces a cellulose-rich substrate with increased porosity and accessibility, which is highly favourable for subsequent processing steps. To further enhance hydrolysis efficiency, the synergistic use of multiple enzymes has been extensively investigated [8]. Endoglucanase selectively cleaves internal β−1,4-glycosidic linkages within the amorphous regions of cellulose, whereas xylanase targets residual hemicellulosic components that hinder enzyme accessibility. The coordinated action of these enzymes facilitates effective removal of structural barriers, increases substrate accessibility, and promotes controlled depolymerization without significant disruption of crystalline cellulose domains. Despite these advantages, the integration of MW-assisted organosolv pretreatment with a dual-enzyme system remains insufficiently explored, particularly for biomass sources rich in pectin and hemicellulose such as fruit peels.

Within this context, the present study proposes an integrated approach integrating MW-assisted AA pretreatment with synergistic endoglucanase-xylanase hydrolysis for the efficient production of nanocrystalline cellulose from tangerine peel. This strategy is designed to enhance the selective removal of non-cellulosic components, improve enzyme accessibility through structural loosening and pore development, and reduce reliance on harsh chemical treatments. By coupling rapid, energy-efficient pretreatment with targeted enzymatic depolymerization, the proposed method aims to achieve high yield, controlled morphology and preserved crystallinity of nanocellulose. This work therefore provides a more sustainable and scalable pathway for nanocellulose production, while addressing key limitations associated with conventional extraction techniques.

## Method details

### Materials and chemicals

Tangerine peel (*Citrus unshiu*), which accounts for approximately 40–50 % of the total fruit weight, and contains a significant amount of cellulose (25–40 %), has been applied as the model feedstock for preparation of nanocrystalline cellulose. The raw tangerine peel (TP) biomass, sourced from a local fruit canning facility, was thoroughly washed, oven-dried, milled and sieved to a mesh screen size of 1–2 mm. Endoglucanase FiberCare® (Novozymes A/S, Batch no CGK20074, specific activity of 812.9 U/g) and recombinant xylanase from *Aspergillus oryzae* (Sigma-Aldrich, CAS No X2753, specific activity of 2500 U/g) were used as hydrolytic enzymes. Citrate buffer solution (0.5 M, pH 4.0, Thermo Fisher Scientific, Cat No.: J61249), a solution of citric acid and sodium citrate dihydrate, was used as the buffer in this enzymatic treatment. Acetic acid (CH_3_COOH, ≥ 99 %, CAS No 64–19–7) and sodium azide (NaN3, ≥ 99.5 %, CAS No 26628-22–8) were procured from Sigma Aldrich Co., USA. All chemicals and reagents were of analytical reagent (AR) grade, and were used as received unless otherwise specified.

### Preparation of nanocrystalline cellulose

**Fig. 1:**
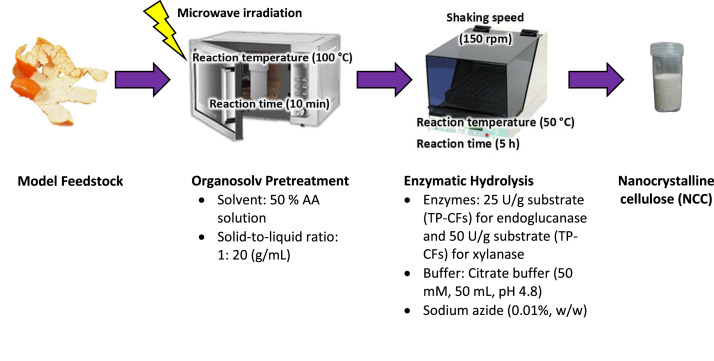
Schematic representation of the TP-NCC synthesis process.

A two-stage approach, incorporating MW-assisted AA pretreatment coupled with enzymatic hydrolysis using a binary endoglucanase/ xylanase mixture, was adopted for the preparation of TP-derived nanocrystalline cellulose (TP-NCC). A schematic representation of the TP-NCC synthesis process is presented in Fig. 1.

### Microwave (MW)-assisted acetic acid (AA) pretreatment

#### Major aim

Organosolv pretreatment facilitates the disruption of biomass cell wall architecture and selective solubilization of lignin, hemicellulose and pectin fractions from lignocellulosic materials. Among organic solvents, AA offers additional merits over mineral acids, in the sense that:

(a) AA exhibits high solvency for lignin, with a Hildebrand solubility parameter (δ value) of 25.8 (J/cm^3^)^−1/2^, close to that of lignin (δ value = 22.5 (J/cm^3^)^−1/2^) and d-polygalacturonic acid (δ value = 31.3 (J/cm^3^)^−1/2^). It also supports hydrogen bond formation with lignin and pectin fragments, resulting in enhanced solubility and delignification efficiency,
(b) AA delignification can be carried out at elevated temperatures (>100 °C) without the need for catalysts, and
(c) AA may be efficiently recovered through simple distillation, which renders the process more environmentally friendly than conventional methods.

#### Procedure

Prior to pretreatment, raw TP biomass (1 g) was swollen in 20 mL of deionized (DI) water (solid-to-liquid ratio = 1:20 g/mL) under continuous mechanical stirring (IKA® RCT Basic Magnetic Stirrer, 3810001, Germany) at 300 rpm and 30 °C for 30 min to remove surface impurities and non-cellulosic particles by flotation. Following this step, the water-swollen biomass was vacuum-filtered to remove excess and unabsorbed water, and the resulting solid material was collected for organosolv pretreatment.

MW-assisted AA pretreatment was conducted in a 50 mL screw-sealed Teflon pressure reactor, and heated using a self-modified microwave oven [Model: Samsung ME711K, Inner dimensions: 36 cm (L) × 33 cm (W) × 24 cm (H)], operating at a power density of 1000 W and a frequency of 2.45 GHz. The microwave setup was upgraded with digital timer-temperature control system, and a high-temperature Type-K thermocouple to enable precise monitoring of irradiation time and temperature throughout the microwave heating. The pretreatment process was conducted by mixing swollen TP sample (1 g) with 50 % AA solution (solid-to-liquid ratio of 1: 20 g/mL). The process was irradiated at 100 °C for 10 min under microwave heating. After completion of the reaction, the reactor was cooled to room temperature within the microwave chamber, and the resulting pulp was filtered and washed repeatedly with deionized (DI) water until a neutral pH (6–7) was achieved. The delignified slurry (TP-CFs) was centrifuged at 6000 rpm for 15 min, and stored at 4 °C for the subsequent enzymatic hydrolysis.

#### Observation

(a) **Colour:** The raw TP slurry exhibited a coarse texture and orange-brown coloration. After MW-assisted AA pretreatment, the reaction mixture turned turbid and darker brown, indicative of phenolic, pectin, hemicellulose and lignin solubilization from the TP matrix. Concurrently, the pre-treated solid residue (TP-CFs) underwent a progressive color transition from orange-brown to light yellow over successive water washing cycles.
(b) **Texture:** The TP-CFs appeared swollen, softened and structurally loosened, with a more pronounced fibrous morphology, characterized by visible separation, detachment and delamination among fiber layers.
(c) **Viscosity:** The slurry became less viscous, due to the removal of lignin, hemicellulose and pectin from the cell walls of TP.
(d) **Odour:** The release of volatile citrus oil produced a strong vinegar-like odor after the heating process.

#### Importance

MW-assisted AA pretreatment is designed to disrupt the recalcitrant structure of lignocellulosic matrix, by partial removal of lignin, hemicellulose, pectin and other non-cellulosic components, and cleavage of hydrogen bonds, van der Waals forces and ester linkages between these chemical constituents. Microwave irradiation provides multiple benefits, including rapid and uniform volumetric heating, improved thermal efficiency, decreased processing time, lower energy usage, and enhanced yield and product consistency [9]. This microwave dielectric heating system accelerates cell walls deconstruction and valorization, and induces steam-filled microfractures within TP-CFs, leading to enhanced enzymatic access to both outer and inner domains of the cellulose fibers. In addition, the use of organic AA in the pretreatment process generates minimal hazardous chemical by-products, while preserving fiber integrity and quality, which is efficient in reducing environmental and technical burdens compared to the chemical counterparts.

### Endoglucanase-xylanase-mediated enzymatic hydrolysis

#### Main aim

The primary objective of mixed-enzyme hydrolysis is to establish a sustainable and selective top-down strategy for the conversion of lignocellulosic biomass into nanocrystalline cellulose, while minimizing chemical use. This approach maximizes cellulose accessibility and hydrolysis efficiency through the coordinated action of enzymes with complementary catalytic functions, without the use of harsh chemical treatments.

Endoglucanase selectively depolymerizes the amorphous regions of cellulose through cleavage of internal β−1,4-glycosidic bonds and disruption of intermolecular hydrogen bonds, reducing the degree of polymerization without extensive degradation of crystalline cellulose. Concurrently, xylanase removes residual hemicellulosic components, particularly xylans, that hinder enzyme penetration and limit cellulose surface exposure. The removal of these non-cellulosic barriers increases pore development and surface area, which promotes improved enzyme diffusion and more homogeneous enzyme-cellulose interactions [10].

The overarching aim of this synergistic enzymatic system is to achieve selective hydrolysis under mild reaction conditions, while preserving the structural integrity of crystalline cellulose domains. Efficient degradation of amorphous cellulose in conjunction with targeted hemicellulose removal allows controlled isolation of nanocrystalline cellulose with reduced energy demand and improved environmental compatibility.

#### Procedure

The enzymatic hydrolysis of nanocrystalline cellulose was conducted in a 100 mL screw-capped Erlenmeyer flask containing 2 % (w/w, dry substrate basis) delignified TP-CFs, 50 mM citrate buffer (adjusted to pH 4.8, measured using a multi-channel benchtop pH meter, Accumet XL 600, Thermo Fisher Scientific) and 0.01 % (w/w) sodium azide to inhibit microbial growth and contamination, with a total reaction volume of 50 mL. The enzyme loadings were 25 U/g substrate (TP-CFs) for endoglucanase and 50 U/g substrate (TP-CFs) for xylanase, which were introduced into the reaction mixture. Prior to hydrolysis, the suspension was homogenized using an ultrasonic bath (FB15057 Fisherbrand® Ultrasonicator) at 25 °C for 30 min to enhance fiber dispersion and enzyme accessibility. The mixture was subsequently incubated in a shaker incubator (Model NB-205, N-biotek, Korea) at 150 rpm and 50 °C for 5 h to ensure uniform enzyme distribution and facilitate selective degradation of amorphous cellulose domains.

At the end of the incubation period (5 h), the enzymatic hydrolysis was quenched by boiling the reaction mixture at 100 °C for 5 min to achieve enzyme inactivation, subsequent by filtration and centrifugation (6000 rpm, 15 min) for the separation of solid and liquid fractions. An aliquot of the liquid fraction (hydrolysate) was withdrawn for total reducing sugar analysis using the 3,5-dinitrosalicylic acid (DNS) colorimetric assay according to Miller’s method [11], whereas the resulting milky-white precipitate was resuspended and washed extensively with DI water by repeated centrifugation under the same conditions until neutral pH (6–7) to remove residual solubilized sugars and denatured enzymes. The final product was homogenized by sonication for 10 min, and oven-dried at 50 °C in an air-circulation oven to constant weight before further characterization.

#### Observation

(a) **Colour:** The light-yellow pulp transitioned towards a near-white colour as the enzymatic treatment removed most amorphous cellulose components. Accumulation of reducing sugars in the liquid phase turned the supernatant from colourless to dark brown, which was evidenced by pronounced colour development in the DNS assay.
(b) **Texture:** The suspension exhibited a soft, gel-like fibrous texture, indicative of the homogeneous dispersion of fine fibrillar fragments.
(c) **Viscosity:** The viscosity of the suspension decreased as large fiber bundles and polysaccharides were broken down into finer and thinner cellulose filaments, reducing fiber rigidity and fiber diameter/ length, as observed in typical fibrillated cellulose systems.
(d) **Odour:** A mild fruity scent was detectable, resulting from the release of soluble sugars or citrus compounds from the cellulose materials.

#### Importance

Biological enzymatic hydrolysis represents a highly sustainable and precise approach for the preparation of nanocrystalline cellulose from lignocellulosic biomass, with greater selectivity and environmental compatibility than conventional chemical approaches. The process operates under mild conditions, minimizes energy and water consumption, lowers chemical input and allows straightforward product recovery, making it inherently scalable [12]. Sequential application of multiple enzymes leverages complementary catalytic mechanisms, enhances cellulose accessibility and hydrolysis efficiency, and preserves the structural integrity and crystallinity of cellulose nanocrystals.

Xylanase plays a critical role by breaking down residual hemicellulose, xylan-lignin complexes and resistant xylan coatings, and converts these components into soluble oligosaccharides. This action opens the cellulose matrix for subsequent enzymatic attack. Endoglucanase acts synergistically to cleave internal β−1,4-glycosidic bonds along cellulose chains and depolymerizes amorphous regions without generating excessive soluble sugars or damaging crystalline domains. The coordinated action of these enzymes creates synergistic effects that exceed additive improvements, achieves high hydrolysis efficiency at moderate enzyme loadings under mild conditions, and preserves nanocellulose morphology, surface chemistry and crystalline structure [13].

This method provides a robust, environmentally compatible platform for the controlled isolation of high-purity nanocrystalline cellulose. By optimizing substrate accessibility, enzymatic efficiency and structural preservation, mixed-enzyme hydrolysis delivers high-quality nanocellulose suitable for advanced materials, bio-composites and industrial applications, positioning it as a benchmark strategy for sustainable cellulose valorisation.

### Technical considerations and precautions in the preparation process

(a) **Microwave equipment:** Microwave heating was conducted using a Teflon-lined reactor. During microwave irradiation, the reactor filling volume should not exceed 60 % of its total capacity, and the reactor cap must be securely sealed/ closed to prevent pressure loss and chemical leakage. Exceeding the recommended loading limit can adversely affect heat transfer uniformity and microwave energy distribution, ultimately diminishing the overall reaction efficiency.
(b) **Incubation operation:** The shaker incubator should be properly calibrated to ensure uniform mixing and consistent reaction conditions, while minimizing sedimentation during incubation. The viscosity of the reaction mixture and the formation of visible foam should be monitored throughout the enzymatic reaction to avoid excessive degradation of cellulosic substrates and enzyme activity.
(c) **Homogeneous dispersion:** Homogeneous dispersion of the reaction mixture was ensured by ultrasonication prior to each treatment step to promote uniform hydrolysis, and limit agglomeration and excessive cellulose degradation. The ultrasonic bath was securely positioned and carefully controlled to minimize splashing or aerosol formation.
(d) **Control of temperature:**
(i) The catalytic effect of acetic acid during pretreatment is significantly intensified at higher temperatures. Microwave irradiation can elevate the reaction temperature to approximately 100 °C, generating substantial internal pressure within the Teflon-lined reactor as a result of acetic acid volatilization. To reduce the risk of inhalation and thermal exposure to acetic acid vapors, the Teflon reactor should not be withdrawn immediately after the microwave heating cycle for sufficient cooling to room temperature.
(ii) Enzymatic reactions are highly temperature sensitive. Therefore, reaction conditions were maintained within the optimal temperature range (∼50 °C) to prevent enzyme denaturation, and to ensure consistent hydrolysis efficiency and reproducibility.
(e) **Optimal pH:** Enzymatic hydrolysis was conducted under controlled pH conditions compatible with both endoglucanase and xylanase. Maintaining the reaction pH within the range of 4.0–5.0 ensured sustained enzymatic activity and reduced the risk of enzyme inactivation or denaturation.
(f) **Optimal reactant dosage and ratio:** Consistent control of chemical reagents, enzymes and substrate loadings is crucial for reproducible extraction. The relative proportion of endoglucanase and xylanase in the mixed enzyme system must be optimized to achieve maximal synergistic hydrolysis. A combination in which xylanase activity equals or exceeds that of endoglucanase has been shown to produce nanocellulose with enhanced key physico-chemical properties. Enzymes must be stored and handled according to the manufacturer’s guidelines to preserve their activities.
(g) **Reaction termination:** The hydrolysis must be quenched promptly to avoid over-hydrolysis, and excessive formation of inhibitory compounds and soluble sugars. The AA pretreatment reaction is terminated with 10-fold volume of cold deionized water to prevent sample carbonization and formation of black residues. Enzymatic reactions are terminated by heating to 100 °C to inactivate the enzymes and suppress further hydrolysis, which could reduce the sample yield.
(h) **Neutralization and washing:** After each nanocellulose extraction stage, the obtained precipitate should be washed thoroughly with DI water until neutral pH (6–7) is achieved to remove residual chemicals, water-soluble components, inhibitory compounds and denatured enzymes, that could interfere with subsequent analyses. All equipment must be carefully cleaned and sterilized after hydrolysis to minimize the risk of cross-contamination in further extraction cycles.

## Method validation

In order to substantiate the effectiveness of the enzymatic preparation methodology, the resulting nanocellulose was subjected to comprehensive physicochemical characterization. The analytical framework comprised evaluation of chemical composition, synthesis yield, total reducing sugar concentration, nanostructural features, crystallinity, functional group identification, thermal behavior, and colloidal stability as indicated by zeta potential (ζ). The specific procedures and instrumentation associated with each method are summarized in Table 1.

**Table 1:** The specific procedures and instrumentation associated with each characterization method.

Characterization methods	Functions	Procedures
Chemical composition	Qualitative identification and quantitative analysis of the chemical constituents	• Technical Association of Pulp and Paper Industry standard protocols: T222 OS-83 for lignin T203 OS-74 for cellulose T249 CM-85 for hemicelluloses • Acid hydrolysis–alcohol precipitation technique for pectin extraction
Production yield	Quantitative measurement of the product generated from a process or reaction, serving as an indicator of the efficiency and effectiveness of the production system	• The production yield (Y) was evaluated using a gravimetric method • The ratio of mass difference between the dry weights of cellulosic samples before (TP-CFs, M_1_) and after the enzymatic hydrolysis (TP-NCC, M_2_) • $Y\left(\%\right)=\frac{M_{2}}{M_{1}}\times 100$
Total reducing sugar	Analytical determination of reducing sugar concentration in the sample matrix	• 3,5-dinitrosalicylic acid (DNS) method • UV–Visible spectrophotometric analysis at 540 nm
Scanning electron microscopy (SEM)	Evaluation of topographical surface attributes, structural features, and dimensional and geometric properties	• A ZEISS Supra 35VP field-emission scanning electron microscope was employed for imaging, operating at 10 kV acceleration potential and 200 pA beam intensity • Sonicated nanocellulose (∼0.001 %, w/v) was drop-cast and sputter-coated with Au prior to imaging
Transmission electron microscopy (TEM)	Examination of particle size distribution, morphological characteristics, and aggregation-dispersion behavior	• A Libra 120 energy-filtered transmission electron microscope (Carl Zeiss) was applied for imaging at an accelerating voltage of 120 kV • Sonicated nanocellulose (∼0.001 %, w/v) was drop-cast onto carbon-coated copper grids, and negatively stained with 2 % ethanolic uranyl acetate • Particle size distribution, particle diameter (D), length (L) and aspect ratio (L/D) were quantified from the average of 100 measurements using ImageJ processing software
X-ray diffraction (XRD)	Determination of crystal structure, phase identification, and orientation and crystallinity index	• XRD patterns acquired using a Bruker-AXS D8 diffractometer at 30 kV and 10 mA •X-ray diffraction patterns were recorded using a monochromatic Cu Kα source (λ = 1.5406 Å) • The diffraction angle (2θ) was scanned from 10° to 80° at a constant rate of 2° per minute, with data points collected at 0.03° intervals •X-ray diffraction data were analyzed using PANalytical X’Pert HighScore Plus software, which incorporates the ICDD database for phase identification and peak matching • XRD-based crystallinity was quantified using a deconvolution method for separating crystalline and amorphous contributions, coupled with Fourier series analysis
Fourier-transform infrared spectroscopy (FT-IR)	Surface chemical characterization and functional assessment	• Fourier-transform infrared spectra were recorded using a PerkinElmer FTIR-2000 spectrophotometer • FT-IR samples were prepared using the pressed KBr pellet method with a sample-to-KBr mass ratio of 1:100 (w/w) • Infrared absorption spectra were measured in transmittance mode between 4000 and 400 cm⁻¹, with a spectral resolution of 4 cm⁻¹
Thermogravimetric analysis (TGA)	Temperature-dependent weight change characterization	• Thermogravimetric analysis was conducted using a PerkinElmer TGA 7 system at a constant heating rate of 5 °C min⁻¹ under an inert nitrogen atmosphere with a gas flow rate of 10 mL min⁻¹ • The TGA curve, depicting mass loss versus temperature, was measured within a temperature window of 50–600 °C
Zeta potential (ζ) measurement	Indicator of particle surface charge and dispersion stability	• Zeta potential measurements were performed using a Malvern Zetasizer Nano-ZS system • A diluted sonicated nanocellulose dispersion (∼0.01 %, w/v) was prepared, and its refractive index (n = 1.43) was determined at ambient temperature using a digital refractometer

The successful synthesis of tangerine peel-derived nanocellulose (TP-NCC) was achieved via the integration of hybrid MW-assisted AA pretreatment with endoglucanase-xylanase-mediated enzymatic processing. A comprehensive validation of the physicochemical attributes of TP-NCC is presented in Table 2, and Table 3 details the chemical composition changes associated with the nanocellulose processing procedures. The green-synthesized TP-NCC exhibited a highly interconnected network of elongated nanofibrils, characterized by an average diameter of 9.23 nm, a visible length of 603.49 nm, and an aspect ratio of 65.36 (Table 2). These dimensional parameters are consistent with the established geometric criteria for cellulose nanofibers, which typically exhibit lengths of 0.5–1.5 µm, diameters ranging from 5 to 30 nm, and aspect ratios ≥ 50 [14].

**Table 2:** Method validation in viewpoints of the morphological, chemical and thermal aspects of TP-derived nanocrystalline cellulose [15].

Properties	TP-NCC	
**Morphology**	(a) SEM image of TP-NCC	Shape: Fibrillar
	(b) TEM micrograph of TP-NCC	Mean length (nm): 603.48 Mean diameter (nm): 9.23 Aspect ratio: 65.36
**Crystallinity**	(c) XRD diffraction patterns of TP-NCC and ICDD’s Cellulose Iβ	Crystallinity index (%): 64.35/ Cellulose Iβ
**Functionality**	(d) FTIR spectrum of TP-NCC	Surface functional group: Hydroxyl group
**Production yield**	Yield (%)	: 70.80
**Thermostability**	Temperature of maximum mass decomposition rates (T_max,_ °C)	: 338.90
**Surface charge**	Zeta potential (mV)	: −30.20

**Table 3:** Chemical compositions of TP, TP-CFs and TP-NCC.

Property Sample	Chemical composition (wt%)				
	Cellulose	Hemicellulose	Lignin	Pectin	Miscellaneous
**TP**	30.82	13.61	11.20	23.56	20.81
**TP-CFs**	61.33	9.87	6.55	7.09	15.16
**TP-NCC**	92.15	0.31	0.16	0.01	7.37

The pronounced fibrillated morphology highlights the synergistic role of MW-assisted AA pretreatment and endoglucanase-xylanase enzymatic hydrolysis in disrupting the recalcitrant supramolecular assembly of tangerine peel and loosening the densely packed cellulose fibril network via a swelling-relaxation mechanism. The synergistic approach markedly enriched the cellulose fingerprint (Table 3) resulting in a production yield of 70.80 %, accompanied by elevated free hydroxyl group content, enhanced crystallinity (*CI* = 64.35 %), improved thermal resistance (T_max_ = 338.90 °C) and a stable negative surface charge (ζ = −30.20 mV), while preserving the native crystalline structure and amphiphilic nature of cellulose Iβ (Table 2).

A comparison of extraction methodologies, and the corresponding nanocellulose yields and thermostability is presented in Table 4. Under optimized reaction conditions and enzyme proportions, the proposed process achieved a markedly higher production yield (70.80 %) and improved physico-chemical characteristics of TP-NCC within a substantially shorter processing time (∼5 h) as compared with conventional nanocellulose preparation techniques. In contrast, the classical conventional chemical production technique exhibited lower production yields (8.9–61.5 %), while the other reported physical-enzymatic routes require longer reaction times (6–72 h). The enhanced performance arises from the synergistic effects of MW-assisted AA pretreatment and endoglucanase-xylanase enzymatic hydrolysis.

**Table 4:** Comparison of extraction methodologies, and the corresponding nanocellulose yields and thermostability.

Precursor	Final product	Pre-treatment		Hydrolysis		Yield (%)	Thermal stability (T_max_, °C)	Reference
		Technique	Temp/ time	Technique	Temp/ time			
Tangerine peel	CNFs	MW-assisted AA pretreatment	100 °C/ 10 min	Endoglucanase + xylanase	50 °C/ 5 h	70.8	338.9	This study
Pineapple leaf fibers	CNCs	MW-assisted NaOH and NaCIO_2_ pretreatment	100 °C/ 90 min, and 80 °C/ 4 h	H_2_SO_4_	45 °C/ 30 min	60.6	263.4	[3]
Bamboo	CNFs	Ball milling	90 min	Endoglucanase + xylanase	50 °C/ 72 h	6.9	-	[4]
Filter paper	CNFs	Homogenization	5 min	Endoglucanase	50 °C/ 24 h	12.4	375.0	[5]
Rice husk	CNWs	MW-assisted NaOH and H_2_O_2_ pretreatment	75 °C/ 70 min	H_2_SO_4_	30 °C/ 10 min	61.5	300.7	[7]
Short-fiber eucalyptus pulp	CNFs	Ball milling	-	Endoglucanase	50 °C/ 24 h	47.7	-	[8]
				Xylanase		8.8	-	
				Endoglucanase + xylanase		68.7	-	
Bleached eucalyptus Kraft pulp	CNWs	Disc ultra-refining	-	Endoglucanase	50 °C/ 72 h	83.1	330.0	[10]
				Xylanase		29.3	339.0	
				Endoglucanase + xylanase		67.2	330.0	
Bleached eucalyptus Kraft pulp	CNFs	Hydrodynamic cavitation	-	Endoglucanase + xylanase	50 °C/ 72 h	60.0	-	[12]
Bleached softwood	CNFs	Neutral-sulfonated treatment/ Parr high-pressure batch reactor	160 °C/ 3 h	Endoglucanase	50 °C/ 6 h	30.1	291.0	[13]
				Endoglucanase + xylanase		37.0	-	
				Endoglucanase + xylanase + mannanase + lytic polysaccharide monooxygenase		40.3	299.0	
Mandarin (*Citrus unshiu*) peel	CNFs	Hydrothermal and HCl	120 °C and 0.12 MPa / 2 h	Sonication	15 s	8.9	-	[16]
Grape pomace	CNCs	H_2_SO_4_ and NaOH pretreatment, and H_2_O_2_/ NaOH bleaching	90 °C/ 5 h and 50 °C/ 8 h	H_2_SO_4_	45 °C/ 30 min	27.6	365.0	[17]
Tomato peel	CNCs	NaClO_2_ bleaching and KOH pretreatment	70 °C/ 5 h and 90 °C/ 2 h	H_2_SO_4_	45 °C/ 30 min	15.7	275.0	[18]
Orange peel	CNFs	NaOH pretreatment and NaClO_2_ bleaching	120 °C/ 30 min and 3 h	Homogenization	-	75.6	250.0	[19]
*Terminalia catappa L*. fruit peel	CNFs	NaOH pretreatment and H_2_O_2_/NaOH bleaching	70 °C/ 3 h and 70 °C/ 3 h	Steam explosion and oxalic acid	121 °C and 15 psi	25.0	372.0	[20]

*CNCs: Cellulose nanocrystals; CNFs: Cellulose nanofibers; CNWs: Cellulose nanowhiskers; H_2_O_2_: Hydrogen peroxide; H2SO4: Sulfuric acid; HCl: Hydrochloric acid; NaCIO_2_: Sodium chlorite; NaOH: Sodium hydroxide.

MW-assisted pretreatment facilitates rapid and uniform heat transfer via dipole rotation and ionic conduction inherent to microwave dielectric heating, and allows AA molecules to align themselves with the oscillating electric field, which ensures efficient dissolution, penetration and better contact with TP [21]. During AA pretreatment, (a) AA would dissociate partially into hydronium (H_3_O^+^) ions, to penetrate into the macro-molecular network/ amorphous region of fibers at elevated temperatures, to be integrated into the nodes -O- and -C = O at C_2_, C_3_ and C_6_ positions of TP, followed by (b) solvolysis or homolytic cleavage of lignin and lignin-carbohydrate bonds, specifically the α-aryl ether and β-aryl ether linkages, and the (c) solvation and dissolution of lignin and pectin fragments in the acid solution, to produce a solid phase that consists of primarily cellulose and hemicellulose [22].

Subsequent enzymatic hydrolysis is enhanced by the coordinated activity of endoglucanase and xylanase, leading to increased substrate accessibility through non-hydrolytic fiber swelling and porosity development, coupled with selective glycosidic bond cleavage, while minimizing disruption of crystalline cellulose regions. Xylanase, as an accessory enzyme in the enzymolysis process, has been shown to both selectively (a) reduce the residual hemicellulose content from the external and internal regions of cellulose fibers by breaking down β−1,4-linkages in the xylan backbone, and (b) convert the relocated/ re-precipitated xylan, xylan-lignin complexes and xylan embedded within the cellulosic matrix into smaller and less recalcitrant fragments. This process generates a more porous and permeable cellulose structure, which enhances the accessibility, penetration and adsorption of exogenous enzymes within both external and internal cellulose fiber regions.

Meanwhile, endoglucanase plays a crucial complementary role which selectively catalyzes accessible intra-molecular β−1,4-glycosidic linkages in the amorphous regions of cellulose and C_1_-O-C_4_ bonds within the cellulose chains, resulting in structurally less-ordered cellulose molecule chains, and newly formed reducing and non-reducing end groups. Overall, the integrated MW-assisted AA pretreatment and enzymatic hydrolysis approach demonstrates superior efficacy relative to conventional methods, in terms of yield, efficiency and structural preservation, without the use of aggressive chemical reagents, highlighting its suitability for sustainable and high-performance nanocellulose production.

### Limitations

(a) **Variability in raw material composition:** The chemical composition of TP biomass is influenced by fruit variety, geographical origin and cultivation conditions, including soil quality, water availability and harvesting time. Variations in cellulose, hemicellulose, lignin and pectin contents can markedly affect pretreatment and enzymatic hydrolysis efficiency, leading to fluctuations in nanocellulose yield, purity and uniformity.
(b) **Risk of over-degradation:** Prolonged exposure time to microwave irradiation and high concentrations of chemical reagents may induce excessive degradation and chain scission of cellulose chains, adversely affecting the physico-chemical characteristics and functional performance of the produced nanocellulose.
(c) **Sensitivity of enzymatic hydrolysis to process conditions:** The effectiveness of enzymatic hydrolysis is determined by multiple factors, including enzyme and substrate concentrations, reaction temperature, pH, duration, agitation intensity and the presence of inhibitory or acceleratory compounds. Systematic optimization of these variables is essential to preserve enzyme activity, regulate inhibitor accumulation, achieve complete fibrillation and produce high-yield, uniform nanocellulose. The slow reaction kinetics, however, can extend processing time and operational costs, potentially limiting the scalability and industrial applicability of the process.
(d) **Environmental considerations:** The use of concentrated acetic acid for pretreatment and sodium azide for enzyme preservation raises environmental concerns. Neutralization of acidic wash water and safe disposal of toxic preservatives are essential to minimize environmental and toxicological impacts.
(e) **High enzyme cost and recovery limitations:** The high cost of hydrolytic enzymes remains a major constraint for industrial-scale enzymatic nanocellulose production. Enzyme performance depends strongly on process conditions, including pH, temperature and ionic strength, which determine catalytic stability and active-site integrity. In lignocellulosic systems, additional activity losses occur due to irreversible enzyme denaturation, non-productive adsorption onto lignin and cellulose surfaces, and incomplete phase separation. These factors reduce effective enzyme turnover and increase operational cost per unit product.

To mitigate these limitations, membrane-assisted enzyme recovery systems have been widely investigated. Integrated microfiltration–ultrafiltration configurations enable both solid–liquid separation and enzyme fractionation. In the first stage, microfiltration (0.22 µm) operates under cross-flow hydrodynamic conditions, and removes insoluble fibrous residues and colloidal particulates. This suppresses cake-layer formation and reduces concentration polarization in downstream membranes. In the second stage, ultrafiltration (10 kDa molecular weight cut-off) enables size-based separation, where high-molecular-weight enzymes such as cellulases (40–80 kDa) and xylanases (20–40 kDa) are retained in the retentate, while low-molecular-weight sugars and oligosaccharides (≤1 kDa) pass through the permeate. The retained enzyme fraction can be recycled through retentate recirculation, which reduces fresh enzyme demand and improves process mass efficiency.

Despite these advantages, long-term operation is limited by progressive enzyme activity loss, irreversible adsorption on membrane and substrate surfaces, and membrane fouling, including pore blocking and gel-layer formation. These issues increase transmembrane pressure and cleaning frequency, and raise both capital and operational costs, which remain key barriers to full-scale implementation.

## Ethics statements

No ethic statements to declare.

## CRediT authorship contribution statement

**K.Y. Lim:** Writing – original draft, Visualization, Methodology, Formal analysis, Data curation. **K.Y. Foo:** Writing – review & editing, Validation, Supervision, Resources, Project administration, Funding acquisition, Conceptualization.

## Declaration of competing interest

The authors declare that they have no known competing financial interests or personal relationships that could have appeared to influence the work reported in this paper.

## Data Availability

Data will be made available on request.
